# Short-term EEG dynamics and neural generators evoked by navigational images

**DOI:** 10.1371/journal.pone.0178817

**Published:** 2017-06-20

**Authors:** Axelle Leroy, Carlos Cevallos, Ana-Maria Cebolla, Stéphanie Caharel, Bernard Dan, Guy Cheron

**Affiliations:** 1 Laboratory of Neurophysiology and Movement Biomechanics, ULB Neuroscience Institut, Université Libre de Bruxelles, Brussels, Belgium; 2 Haute Ecole Condorcet, Mons, Belgium; 3 Departamento de Ingeniería Mecánica, Facultad de Ingeniería Mecánica, Escuela Politécnica Nacional, Quito, Ecuador; 4 Laboratoire de Psychologie de l’interaction et des relations intersubjectives (InterPsy-EA4432), Université de Lorraine, Nancy, France; 5 Inkendaal Rehabilitation Hospital, Vlezenbeek, Belgium; 6 Laboratory of Electrophysiology, Université de Mons-Hainaut, Mons, Belgium; Fondazione Santa Lucia Istituto di Ricovero e Cura a Carattere Scientifico, ITALY

## Abstract

The ecological environment offered by virtual reality is primarily supported by visual information. The different image contents and their rhythmic presentation imply specific bottom-up and top-down processing. Because these processes already occur during passive observation we studied the brain responses evoked by the presentation of specific 3D virtual tunnels with respect to 2D checkerboard. For this, we characterized electroencephalograhy dynamics (EEG), the evoked potentials and related neural generators involved in various visual paradigms. Time-frequency analysis showed modulation of alpha-beta oscillations indicating the presence of stronger prediction and after-effects of the 3D-tunnel with respect to the checkerboard. Whatever the presented image, the generators of the P100 were situated bilaterally in the occipital cortex (BA18, BA19) and in the right inferior temporal cortex (BA20). In checkerboard but not 3D-tunnel presentation, the left fusiform gyrus (BA37) was additionally recruited. P200 generators were situated in the temporal cortex (BA21) and the cerebellum (lobule VI/Crus I) specifically for the checkerboard while the right parahippocampal gyrus (BA36) and the cerebellum (lobule IV/V and IX/X) were involved only during the 3D-tunnel presentation. For both type of image, P300 generators were localized in BA37 but also in BA19, the right BA21 and the cerebellar lobule VI for only the checkerboard and the left BA20-BA21 for only the 3D-tunnel. Stronger P300 delta-theta oscillations recorded in this later situation point to a prevalence of the effect of changing direction over the proper visual content of the 3D-tunnel. The parahippocampal gyrus (BA36) implicated in navigation was also identified when the 3D-tunnel was compared to their scrambled versions, highlighting an action-oriented effect linked to navigational content.

## Introduction

Virtual reality (VR) is increasingly used in both experimental and clinical settings allowing the recording of brain activity inside a controlled ecological environment [1], [2]. Navigation inside a 3D-tunnel [3], [4], [5] a city [6], [7], [8], [9], [10] or a landscape [11] are the most popular applications of VR [1], [2].

Thanks to these approaches, the neural structures involved in path-finding have been identified with different imagery supports [10], [12]. As VR is primarily supported by visual information, the image content immediately imposes a representation supported by specific bottom-up [13] and top-down processing [14], [15], [16]. For example, the recognition of the image of a checkerboard, a 3D-tunnel or a familiar face is performed effortlessly without specific requirement about the behavioral task. The robust human visual recognition processes can recreate complex behaviors from visual inputs alone. A unified percept may emerge from coordinated neuronal networks widely distributed in the brain [17] influenced by the experience relating to image content, sense of minimal self and state of action [18], [19]. In addition, the specificity of VR image content and the related top-down context may influence the anticipation of the subject during action observation [20]. In this context sport experts outperform novices in action anticipation behaviors [21], [22].

New data in cognitive neuroscience highlights the importance of the relationship between cognition and action, emphasizing the role of changing environment and behavioral prediction in neuronal network dynamics involved in perception [23]. The brain uses top-down processing efficiently to anticipate forthcoming sensory events from the environment. Whereas stimulus-driven bottom-up processing sequentially extracts increasingly elaborate representation from the initial percept, top-down, expectation-driven processing involves ‘enslaving’ influence on local neuronal behavior by large scale dynamics [23]. For example, top-down processing documented in selective attention is supported by long range cortico-cortical projections linking the parietal and frontal cortex [24], [25] and the cerebellum to the visual areas [26], [27].

The performance of the visual cortex is related to an efficient combination of bottom-up (local circuitry) and top-down processing (long-range connections) [28], [29]. These neural connections participate in the reentry mechanism [30] that generates oscillatory activity in specific frequency bands directly accessible by electroencephalography (EEG).

Because the top-down processing is dependent of the environment, the study of the EEG activity of astronauts in weightlessness provides insights into top-down influence on the visual perception in the absence of graviceptive information [31]. In particular, marked differences in EEG dynamics were observed between processing of action-related (navigational) image 3D-tunnel, see [32] and classical checkerboard image, suggesting that different neural generators support top-down processing according to image content.

The aim of the present paper is to characterize the EEG dynamics and the related ERP generators implicated in the perception of a classical checkerboard pattern and the 3D-tunnel. We hypothesize that because the 3D-tunnel contains a directional cue, it leads to long-lasting modulation of EEG oscillations implicating different generators linked to navigation from those involved in checkerboard pattern perception.

## Materials and methods

### Participants

The data was collected from 20 healthy volunteers who were distributed into 3 complementary paradigms. All participants were right-handed and had no neurological condition and normal vision, including 3D vision. The first paradigm was performed in 8 participants (2 females and 6 males, mean age: 22 ± 3 years) and the other two in 12 (4 females and 8 males, mean age: 24 ± 3 years) (see below). Participants gave informed consent to take part in the study, which was approved by the local ethics committee of the Brugmann Hospital, Brussels, Belgium.

### Experimental paradigms

**Fig 1:**
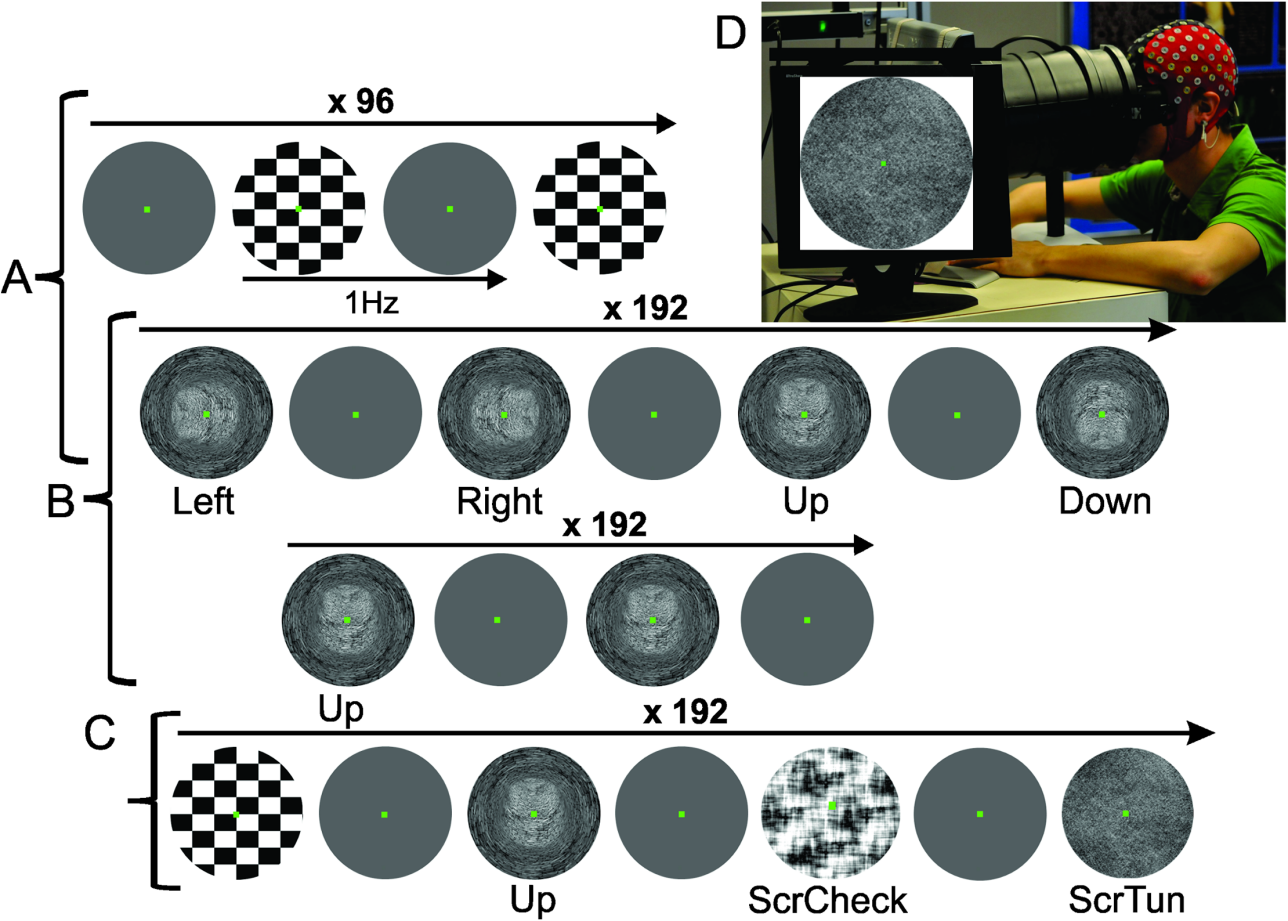
Overview of the three different stimulation paradigms and experimental settings. A: paradigm 1, checkerboard images (upper line) were compared to 3D-tunnel (lower line) (four directions randomly presented, (Left, Right, Up, Down)) intermixed with uniform gray images. Each visual item was presented for 500 ms. 96 and 192 presentations were used for the checkerboard and the 3D-tunnel, respectively. B: paradigm 2, 3D-tunnel (same condition as in A) compared to 3D-tunnel one direction (Up)(lower line) intermixed with uniform gray images. C: paradigm 3, checkerboard, 3D-tunnel (Up) scrambled checkerboard (ScrCheck) and scrambled 3D-tunnel (ScrTun), were randomly presented intermixed with gray image. For all paradigms, a green fixation point was presented on the center screen. The participants were asked to maintain their eyes on a green fixation dot presented centrally. D: Experimental setup. The subject is equipped with an EEG-cap and looks straight-ahead through a form fitting facemask connected through a cylindrical tunnel to laptop screen centered on the line of gaze at a distance of 30 cm from the eyes.

In a first paradigm (n = 8) we presented a sequence of checkerboard comprising 96 images intermixed with 96 gray images and a sequence of 3D-tunnel presentations containing 192 images from four randomized corridor directions (up, down, right and left) giving an implicit illusion of virtual navigation intermixed with gray images (Fig 1A).

In order to assess the influence of the directional cue in the 3D-tunnel a second paradigm (n = 12) was performed with a series of 192 presentations of 3D-tunnel with the same direction (upward) and 192 presentations of 3D-tunnel with the four different directions intermixed with 192 gray images (Fig 1B). In the third paradigm (n = 12) a series of 192 presentations of 3D-tunnel with the same direction (upward) was randomly mixed with 192 scrambled versions of the 3D-tunnel, 192 checkerboard patterns, 192 scrambled versions of the checkerboard intermixed with gray patterns (Fig 1C).

### Stimulation and recording parameters

Participants watched the screen of a laptop straight-ahead through a form-fitting facemask. A video monitor was centered on the line of gaze at a distance of 30 cm from the eyes (Fig 1A–1C). The monitor was viewed through a cylindrical tunnel, thus removing any external visual references (Fig 1D).

### Checkerboard versus 3D-tunnel and scrambled versions

All participants performed passive observation without any type of intention or attention to the visual stimuli. The checkerboard pattern and the 3D-tunnel were alternatively presented with a uniform gray image (Fig 1) on the EGA screen of an IBM Laptop (screen of 22.0 cm height, 30.3 cm width; refresh rate of 75 Hz, resolution of 800 x 600 pixels). An identical stimulation rate (1.0 Hz) was used in both conditions. Checkerboard stimulus consisted of black and white rectangles (4.5 x 4.0 cm) alternating 96 times with the gray page, which corresponded to full-contrast black and white checkerboard (black field 15 lx; white field 101 lx). The gray page luminance was about 43 lx.

The 3D- tunnel was non-stereoscopic but included perspective cues generated by the OpenGL graphic libraries [33]. It represented a tunnel with stone-textured walls (stone dimension 1.25 cm^2^ at the periphery to 0.15 cm^2^ close to the center) in the form of a pipe with constant circular cross-sections. These different stimuli with a pattern contrast of about 50% display subtended 7°(w) × 5° (h) at the eye. Thus, both foveal and parafoveal retinal fields were stimulated. The luminance of the tunnel evolved from 39 lx at the periphery to 74 lx close to the center. The presentation of each visual item (presentation time of 500 ms) was immediately followed by the presentation of a uniform gray image (also for 500 ms). The scrambled patterns were constructed by phase-scrambling of these images using the Fourier phase randomization procedure [34] where the phase spectrum of the 3D-tunnel and the checkerboard were replaced by random values, maintaining the amplitude spectrum and the global low-level properties of the original images (spatial frequency spectrum, contrast and luminance) [35] while the shape and direction information of the tunnel were lost.

### EEG recordings

The EEG was recorded from 128 scalp sites using a shielded electrocap. All recordings were referenced to the left earlobe electrode. Vertical and horizontal eye movements (EOG) were recorded bipolarly. All electrode impedances were maintained below 5 kΩ. Scalp potentials were amplified by ANT DC-amplifiers (ANT, the Netherlands) and digitized with a rate of 2.048 Hz, a resolution of 16 bit (range 11 mV). A band pass from DC to 256 Hz and a notch filter (47.5–52.5 Hz) were also applied. Participants were asked to avoid eye blinks and to fixate the green dot presented in the middle of the screen in order to reduce eye artifacts. In order to verify the effectiveness of the eye fixation requirement, the number of eye movements was recorded throughout the different visual stimulation periods. For this, saccades including small saccades of about 0.8° and other eye movements were automatically selected by a Matlab (MathWorks Inc) script using eye velocity threshold [36]. This selection was then verified by visual inspection. For all subjects, the fixation requirement was respected. Only 0.19 ± 0.09 saccades per second were recorded and this mean number was not different depending on the different images.

Off-line treatment and statistics were performed by means of EEGLAB software [37] and artifactual portions of the EEG data were rejected after appropriate independent component analysis (ICA).

### Event-related potentials

**Fig 2:**
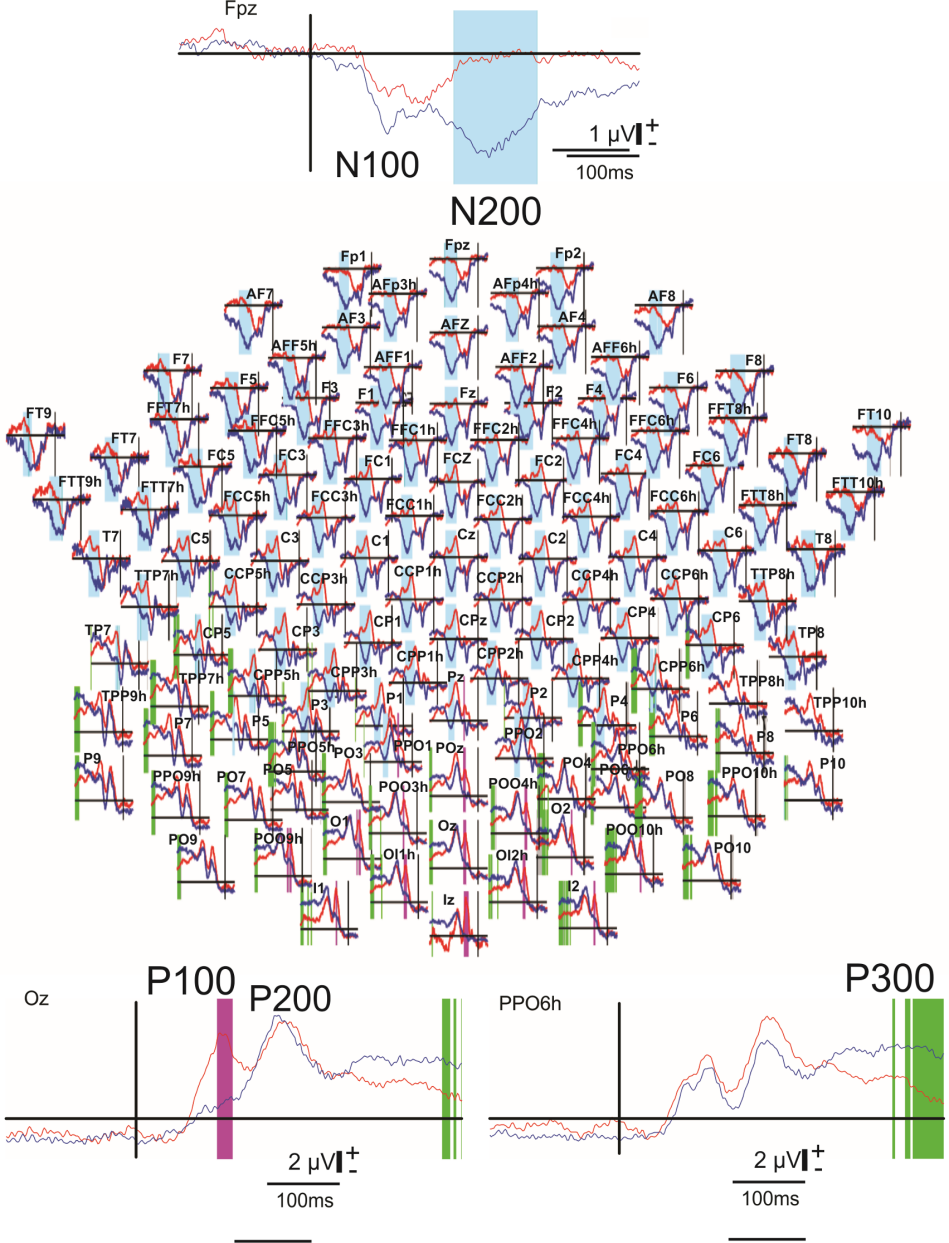
Superimposition of the event-related potentials (ERP) (*paradigm 1*, grand averaged n = 8) corresponding to the presentation of the 3D-tunnel (blue) and checkerboard (red) patterns. Full scalp array of these ERP (128 electrodes, reference placed on the right earlobe)(center). The shaded areas indicate significant difference (*P* < 0.05, permutation test) in the ERP periods between the two conditions: pink, blue and green areas for P100, P200 and P300 periods, respectively. Recordings at the frontal locus (Fpz) highlighting N200 (top insert). Recordings at the occipital locus (Oz) highlighting P100 (left bottom insert). Recordings at the parietal locus (PPO6h) highlighting P300 (right bottom insert). The vertical lines indicate the stimuli onset. Calibration bar corresponds to 2 V, positivity up.

After a global statistical analysis (permutation test and Holms correction) on the full scalp array (see Fig 2), visual evoked potentials (VEP) were measured at the occipital (Oz), parietal (PPO6h) and frontal (Fpz) loci with respect to the reference electrode placed on the left earlobe.

### Event-related spectral perturbation

The EEGLAB software permits to analyze the event-related dynamics and to decipher the ongoing EEG processes that may be partially time- and phase-locked to experimental events [38]. The ERSP may correspond to a narrow-band of event-related desynchronization (ERD) or synchronization (ERS). Briefly, for this calculation, EEGLAB computes the power spectrum over a sliding latency window of 256 ms in each trial and then average across trials. Following the gain model [37], for each frequency band at each frequency point the power spectrum is divided by the averaged spectral power in the pre-stimulus baseline period (-500 ms to 0 ms). The log-transformed of this measure allow the visualization of wider range of variation [39]. Each epoch contained samples from 1.000 ms before and 2.000 ms after the stimulus.

### Inter-trial coherence

ITC is a frequency-domain measure of the partial or exact synchronization of activity at a particular latency and frequency to a set of experimental events to which EEG data trials are time-locked. This measure was also called ‘phase locking factor’ [40]. The ITC measure took values between 0 (no synchronization) and 1 (perfect synchronization). In order to quantify ERSP and ITC with respect to time and frequency, we calculated the ERSP and ITC volume expressed in arbitrary units. In order to increase the time resolution, the time onset and termination of the ERS and ERD, periods were measured by using Morlet wavelet transform [41]. We used wavelet transform for complex spectro-temporal representation with Hanning-windowed sinusoidal wavelets at 1 cycles (lowest) to 12.5 cycles (highest). ERSP and ITC templates were calculated with 200 time points (-721.5 ms to 1221.5 ms), using a window size of 285 samples (556.6 ms) at 97 linear spaced frequencies from 2 to 50 Hz. For the significance level of ERSP and ITC, a bootstrap resampling (p < 0.05) was used as a surrogate method [37]. In this condition, for a Δ*f* of 0.5 Hz the temporal accuracy (Δ*t*) was calculated using the following formula: (Δt=12πΔf) was of 16.3 ms.

The maximal values of ERSP and ITC were measured in the same time frames of the corresponding ERP interval around the peaks.

### Inverse modeling method (swLORETA)

We applied swLORETA (Standardized Weighted Low Resolution Electromagnetic Tomography) [42], [43] as source reconstruction method. It represents a distributed inverse solution method able to model spatially distinct sources of neuronal activities without prior knowledge about the anatomical location of the generators. Derived from the sLORETA method introduced by Pascual-Marqui et al. [44], swLORETA allows to accurately reconstruct surface and deep current sources even in the presence of noise and when two dipoles are simultaneously active. This was realized by incorporating a singular value decomposition based lead-field weighting that compensated for the varying sensitivity of the sensors to current sources at different depths [42]. It must be recognized that swLORETA has spatial resolution limitations in comparison with fMRI. However, contrary to fMRI, EEG is a direct measure of (global) actual electrical brain activity with high temporal resolution allowing swLORETA to delineate the various electric generators within the first 300 ms.

### ERP source analysis

In order to map the generators of the main ERP components evoked by the checkerboard and the 3D-tunnel, we computed swLORETA solutions for the same time periods around the peak of the P100, P200, P300 components in the different conditions. Briefly, the swLORETA solution was computed using a 3D grid of points (or voxels) that represented possible sources of the signal. Based on the probabilistic brain tissue maps provided by the Montreal Neurological Institute (MNI) [45], MNI [46], [45], [47] the solution was restricted to the gray matter and cerebellum by taking only voxels in which the probability of gray matter was unequal zero. Then, the 2030 grid points (5.00-mm grid spacing) and the recording array (128 electrodes) were placed in registration with the Collins 27 MRI produced by the MNI [45]. The Boundary Element Model (BEM) was used for solving the forward problem [48]. The final coordinates (x,y,z, Talairach coordinates) we provided for labeling the corresponding brain areas were based on Talairach atlas. These coordinates were obtained by placing the corresponding Talairach makers in the Collin brain using the ASA software. By this way and by using a Talairach atlas [49], it was possible to identify the corresponding Brodmann areas inside the cortical mantle. The correspondence between the functional zones of the visual cortex mentioned here (V1, V2, V3, V4 and V5/MT) and the Brodmann areas is provided by [50]. For the definition of cerebellar regions we used the nomenclature of the MRI Atlas of the Human Cerebellum of [51].

### Statistical analysis

The statistical significance of difference between the two experimental conditions (“checkerboard” and “3D-tunnel”) amplitude and respective latency in time (ERP) and in time-frequency domain analysis (ERSP and ITC) was calculated with ANOVA (and post-hoc Freeman correction) in the selected EEG channels. For each stimulation, the peak latency and mean amplitude around the peak (ranging from -20 ms to 20 ms) were measured [52] for the components P100, P200 in posterior areas and concomitant N100, N200 in frontal areas and late centro-parietal P300. In order to estimate the effect size (d) on the statistical significance, post hoc power analyses were performed with the G*Power 3.1 software [53]. The power (*1-β)* was computed for all the significant *P* < 0.05. For significance in the full scalp array in the ERP, ERSP and ITC, we employed a nonparametric permutation and the Holm’s method [54] to correct for multiple comparisons. This method is provided by EEGLAB software [37].

To find the generators of the P100, P200 and P300 components, a rigorous method was used to establish a *threshold value* that helps in identifying statistical significance of the current density magnitude. To do so, we used the nonparametric permutation test described in Nichols and Holmes [55]. The rationale for using this method for the inverse solution was explained in details in [43]. In order to use the *t* test as the value of merit, we subtracted one to each voxel. In order to build the empirical distribution for the Holmes method, we randomly multiplied the value of each voxel by -1 or 1. Finally, we performed a total of 8192 *t*-tests for each component analyzed. The normalization process is described by the following formula:

$$J_{i}{\left(t\right)}_{normalized}=\frac{J_{i}(t)}{\sum_{voxel=0}^{nVoxel=2030}J_{voxel}(t)}-1$$

where *J*_*i*_(*t*) represents the inverse solution for the *i*-th voxel for the time *t*.

## Results

### Event-related potentials

A full scalp array of the grand average superimposition of the ERP evoked by the checkerboard (red) or the 3D-tunnel presentation (blue) (Fig 2) highlights the differences obtained in the time domain analysis between these two conditions (*paradigm 1*, Fig 1A). An early significant period (*P* < 0.05, permutation test) around P100 peak (Fig 2, pink areas and left bottom insert) was observed over the occipito-parietal areas, followed by a second period around the P200-N200 latency (Fig 2, blue areas and upper insert) over the central and frontal areas and a third significant period around the P300 peak (Fig 2, green areas and right bottom insert) over the occipito-parietal areas. In order to gain insight in these differences, we have separately studied these ERP periods. We observed a smaller amplitude of the occipital P100 (measured at Oz electrode,) in case of the tunnel (1.98 ± 0.75 μV versus 4.9 ± 2.6 μV, *P* < 0.008, n = 8 subjects (ANOVA), d = 1.52, power = 0.89), a same trend was observed for the frontal N100 (measured at Fpz electrode,) but without reaching statistical difference (2.6 ± 2.1 μV versus 3.0 ± 1.6 μV, *P* = 0.6, n = 8 subjects (ANOVA)). Moreover, the amplitude of the occipital P200 (electrode Oz) was not significantly different for the tunnel (6.2 ± 3.8 μV) than for the checkerboard (5.9 ± 3.6 μV, *P* = 0.8, n = 8 subjects (ANOVA), d = 2.12, power = 0.99), whereas a centro-frontal N200 (electrode Fpz) of 3.6 ± 1.7 μV of amplitude emerged for the 3D-tunnel presentation and was not present for the checkerboard stimulation. This indicates that the classical inversion of polarity between the occipital and frontal loci only existed for P100-N100 in both situations, but not for the P200-N200 elicited by the checkerboard. N200 was recorded in the frontal locus when the 3D-tunnel was presented but its peak latency arrived significantly later than the occipital P200 (251 ± 26 ms for the N200 versus 222 ± 11 ms for the P200, n = 8 subjects, *P* < 0.006 (ANOVA), d = 1.46, power = 0.87).

In addition, the P100 peak latency was longer for the checkerboard than for the 3D- tunnel (122 ± 12 ms versus 101 ± 17 ms, n = 8 subjects, *P* < 0.005 (ANOVA), d = 1.43, power = 0.86). For the P200, the difference in the latency peak between the two types of stimulus followed the same tendency as for P100 with a statistical significance (236 ± 9 ms versus 222 ± 11 ms, n = 8 subjects, *P* < 0.007 (ANOVA), d = 1.39, power = 0.84). The N100 peak latency between the checkerboard and 3D-tunnel stimuli was not statistically different (121 ± 23 ms versus 109 ± 21 ms; respectively, n = 8 subjects, *P* = 0.24 (ANOVA)).

**Fig 3:**
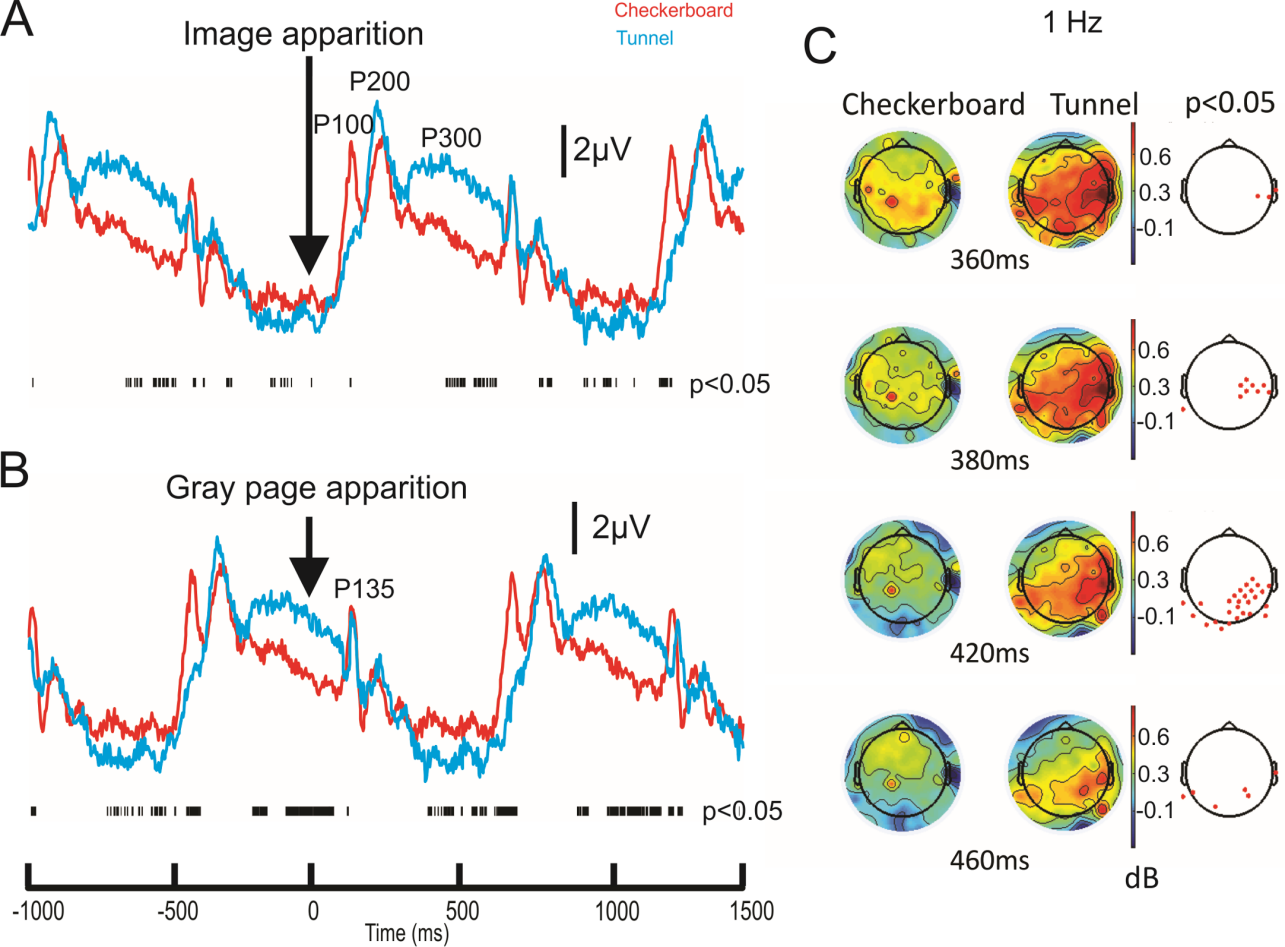
Superimposition of the event-related potentials (grand average, n = 8 subjects) evoked by the checkerboard (red traces) and the 3D-tunnel (blue traces) presentation recorded at the O2 electrode. A: The vertical arrow corresponds to image presentation (Checkerboard or 3D tunnel) highlighting the P100, P200 and P300 components. The lower raster line represents the statistical significance of ERP difference between the two conditions (*P* < 0.05, permutation test). B: Same display as in A, but here the vertical arrow indicates the presentation of the uniform gray image highlighting the P135 component. Note the occurrence of a sinusoidal profile in the delta frequency range. C: Scalp topographies of the power of delta oscillation (1Hz) corresponding to the checkerboard and the 3D-tunnel stimulation between 360 and 460 ms. Only the significant periods are illustrated and the significant electrodes (*P* < 0.05, permutation test) are displayed in the far right column.

Fig 3 illustrates the whole ERP sequences ranging from -1000 to 1500 ms with respect to the checkerboard versus 3D-tunnel (Fig 3A), or the gray image occurrence (Fig 3B). This represented the gray-3D-tunnel/checkerboard-gray and 3D-tunnel/checkerboard-gray-3Dtunnel/checkerboard sequences. We observed in this condition (Fig 3, O2 recording) a sinusoidal profile emerging at about 1.0 Hz. The positive phase of this slow wave followed the presentation of the checkerboard or 3D- tunnel while the negative phase followed the presentation of the gray image. With the exception of P100 period, the two sinusoids were significantly different during the period ranging from 360 to 460 ms; the trace corresponding to the 3D-tunnel (blue trace) presenting an increased amplitude with respect to the red trace corresponding to the checkerboard stimulation. As no clear peaks appeared during this period, we decided to consider it as a P300 related process presenting a higher amplitude (3.53 ± 1.81 μV) for the tunnel than for checkerboard (1.92 ± 1.01 μV, n = 8 subjects, *P* < 0.05 (ANOVA), d = 1.23, power = 0.75). FFT analysis demonstrated that the 3D-tunnel evoked a stronger power in the delta theta band (~ 1Hz) over the right parietal locus and that between 360 and 470 ms (n = 8 subjects, *P* < 0.05 (permutation test) (Fig 3C). The analysis of the ERP evoked by the gray screen showed an absence of difference (latency and amplitude) between the two different conditions in the main positive component peaking at about 135 ms (P135 in Fig 3B).

### Event-related spectral perturbations analysis

**Fig 4:**
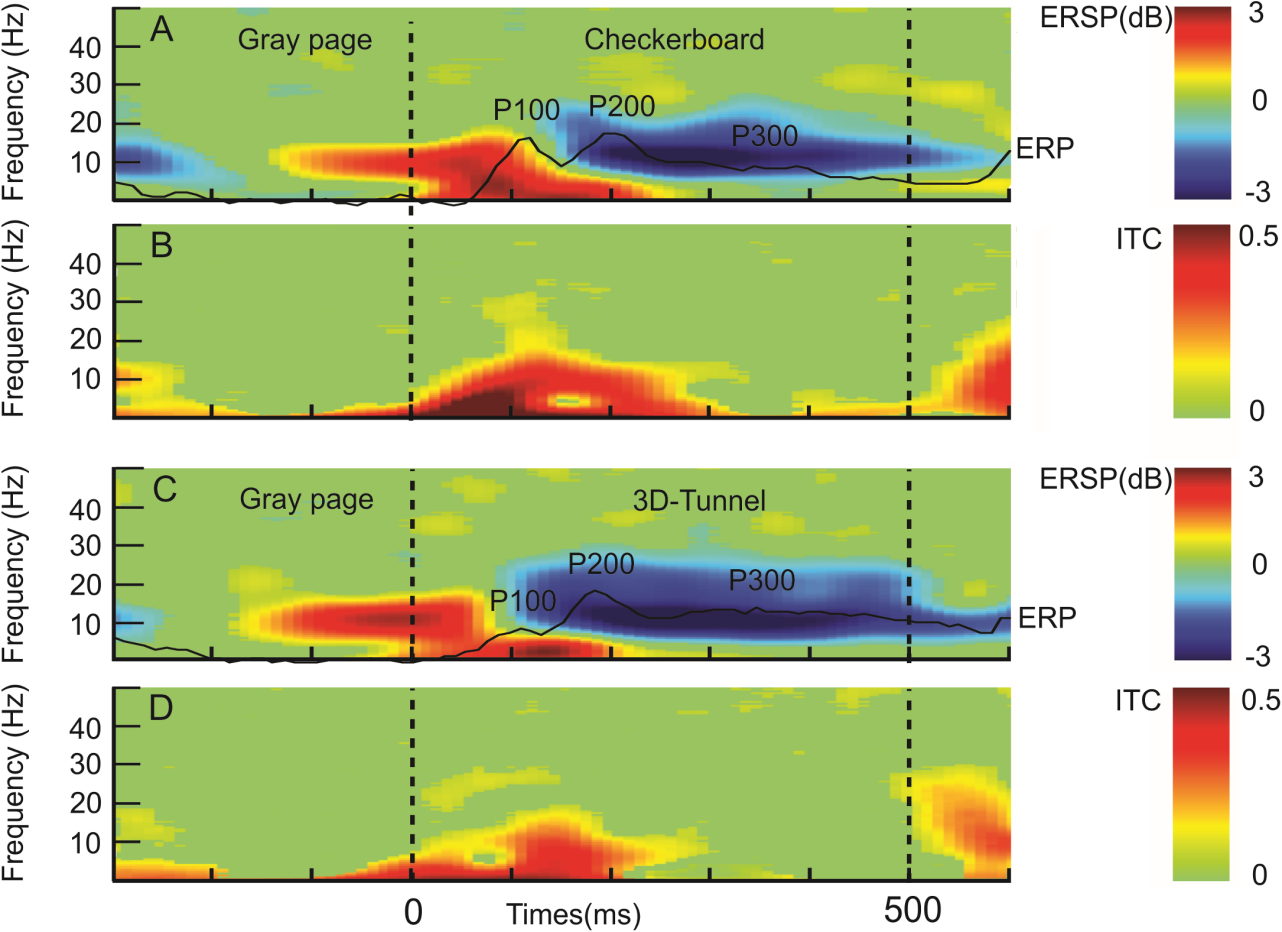
Event-related spectral perturbation (ERSP) and intertrial coherency (ITC) (grand average, n = 8 subjects) recorded at the O2 electrode. A: ERSP related to checkerboard stimulation preceded and followed by gray page condition, the related ERP traces are overlapping and indicate P100, P200, P300. B: ITC map corresponding to the same situation as A. C: Same display as in A but for the 3D tunnel stimulation. D: same display as in B, but for the 3D-tunnel stimulation.

**Fig 5:**
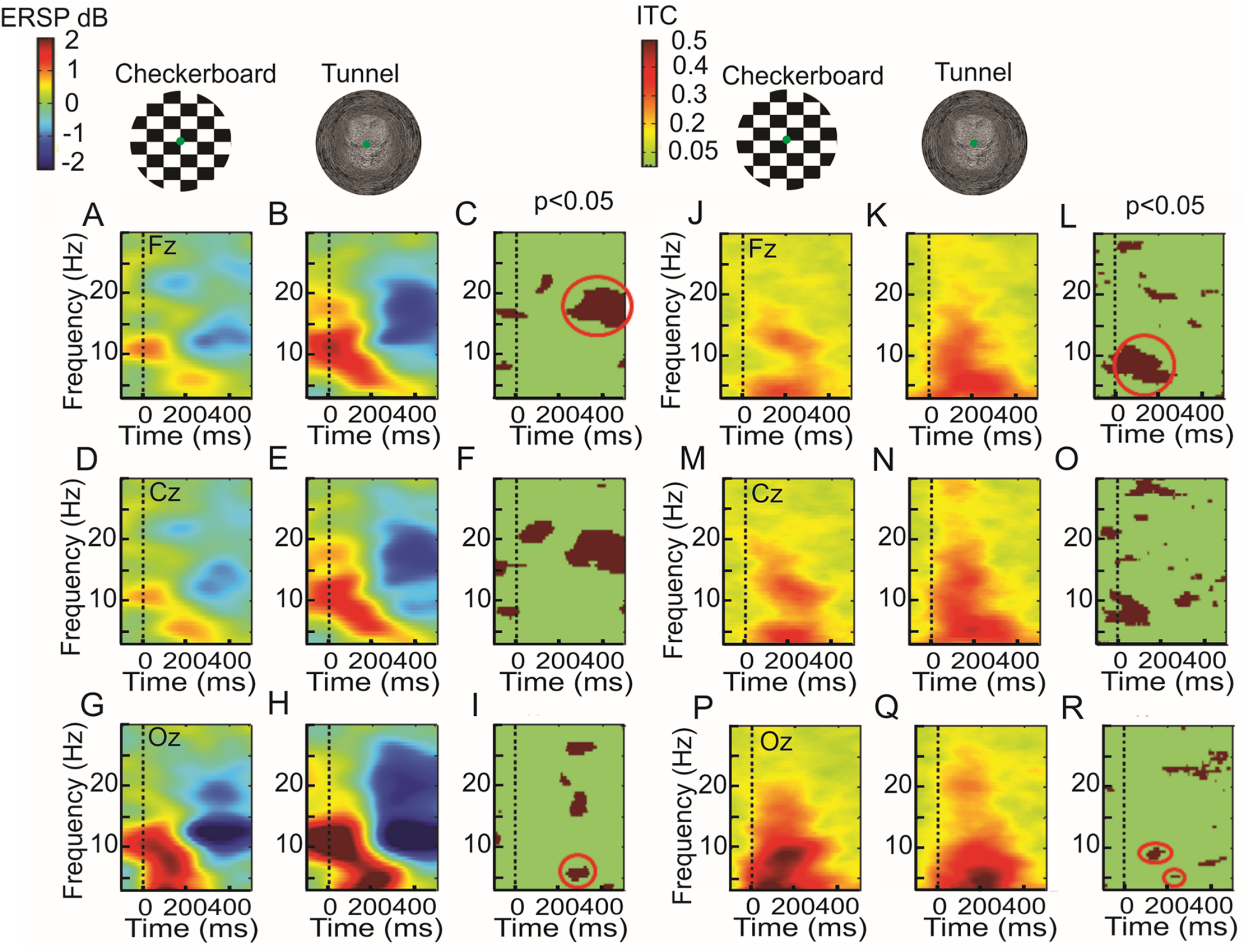
Event-related spectral perturbation (ERSP)(left side)(A-I) and intertrials coherency (ITC) (right side)(J-R)(grand average, n = 8) evoked by checkerboard or 3D-tunnel stimulation. The first line corresponds to the Fz recording, the second line to Cz recording and the third line to Oz recording. The third and sixth columns represent the statistical map (permutation with Holms, *P* < 0.05). The circles emphasize the significant area of the stronger beta ERD for the tunnel at about 400 ms (C), the significant theta ERS for the tunnel at about 300 ms, (I), the stronger alpha ITC for the tunnel (L) in the early period. Note the stronger alpha ITC for the checkerboard at 150 ms and the stronger theta ITC at about 250 ms for the tunnel (R).

**Fig 6:**
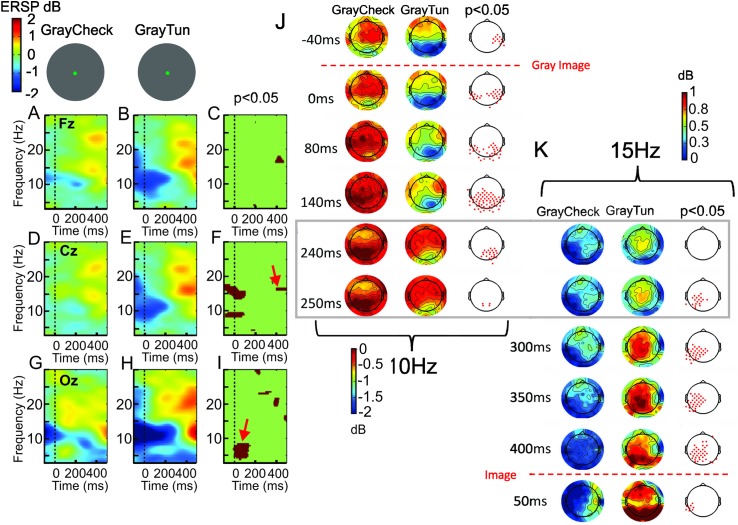
Event-related spectral perturbation (ERSP) evoked by the gray image in the context of the checkerboard (GrayCheck)(A,D,G) and the 3D-tunnel (GrayTun)(B,E,H) in the same display as in Fig 5. The statistical differences observed between these two situations are indicated in C, F, I for the ERSP recorded in Fz, Cz, and Oz, respectively. The red arrows point to increased beta ERS in case of the GrayTun centered around 450 ms (F) and increased alpha ERS just after the onset of the GrayCheck. These ERPS analysis are corroborated by FFT topographies of the 10 Hz (J) and 15 Hz oscillations (K) where only the statistical periods are illustrated from respectively -40 ms before the gray image presentation to + 250 ms and from +240 ms after the gray image to + 50 ms after the image presentation. J and K maps are vertically adjusted in order to highlight the time transition marked by a rectangular frame between the 10 and 15 Hz maps. Note that significant electrodes (third column of J, K) are mainly bilaterally situated in the parieto-occipital areas (J) for the 10 Hz and mainly lateralized in the left side for the 15 Hz (K).

Fig 4 illustrates the grand averaged profile of the ERSP and ITC analysis along time of the succession of the gray image followed by the checkerboard (Fig 4A and 4B) or the 3D-tunnel (Fig 4C and 4D) and the gray image again (*paradigm 1*, Fig 1A). This allowed us to highlight the whole temporal extension of the ERSP and ITC in function of the checkerboard or the tunnel presentation. The alpha-beta ERS occurred before the image presentation. However, at the latency of about -190 ms the ERS amplitude was stronger for the 3D-tunnel than for the checkerboard (*P* < 0.05, permutation test n = 8 subjects) (Figs 5F and 6F). This indicates that the anticipation of the 3D-tunnel was stronger than for the checkerboard. In the same line of evidence, the temporal extension of the alpha-beta ERD after the end of the presentation was stronger in case of the 3D-tunnel with respect to the checkerboard (ERD duration of 225 ± 86 ms for tunnel versus 111 ± 53 ms for the checkerboard, n = 8 subjects, *P* < 0.01 (ANOVA), d = 1.59, power = 0.92).

Fig 5 illustrates the comparison of the ERSP and the ITC maps related to the checkerboard and the 3D-tunnel presentation for the frontal, central and occipital loci. At the latency of P100, the ERSP analysis showed that the 3D-tunnel evoked a stronger ERS (Fig 5H) in the alpha-beta band (~15 Hz) with respect to the checkerboard pattern (Fig 5G) (1.94 ± 1.24 dB versus 0.81 ± 0.48 dB; n = 8 subjects *P* < 0.032 (ANOVA), d = 1.2, power = 0.99) this significance was confirmed for the central (Fig 5F) and frontal (Fig 5C) loci with the permutation test but not for the occipital one (Fig 5I). At the occipital locus, the ITC in alpha band was statistically stronger at the latency of about 140 ms for the checkerboard image (0.58 ± 0.13) (Fig 5P) than for the tunnel (0.39 ± 0.08)(Fig 5Q)(n = 8 subjects, *P* < 0.02 (ANOVA), d = 1.76, power = 0.95). For the occipital theta ITC, the maximal value at about the respective P100 latencies was stronger for the checkerboard (0.52 ± 0.07 versus 0.37 ± 0.11, respectively, n = 8 subjects *P* < 0.007 (ANOVA), d = 1.62, power = 0.92). We observed the inverse situation for the occipital theta ITC at about the respective latencies of P200 (Fig 5P–5R) where the ITC max was stronger for the 3D-tunnel (0.59 ± 0.15) than for the checkerboard (0.37 ± 0.15) (n = 8 subjects, *P* < 0.01 (ANOVA), d = 1.47, power = 0.87). It is interesting to note that although no task was asked to the subject (passive viewing), the ERSP map showed the presence of a significant ERD extending from the alpha towards the upper beta band with an onset at about 250 ms (Fig 5B, 5E and 5H) and ending well after the arrival of the gray image (Fig 6B, 6E and 6H). This ERD was concomitant to the presence of a P300 process as previously reported [56,57]. The frequency range of this ERD extended from 8 Hz to 28 Hz, the maximal amplitude of the ERD (ERD_max_) was observed in the upper alpha band (~13 Hz) for the occipital locus and in the beta band (~17 Hz) for the central and frontal loci. In the frontal locus beta ERD_max_ peaked at 437 ± 27 ms in case of the checkerboard stimulation and at 439 ± 10 ms for the 3D-tunnel and presented a significant difference in amplitude in favor of this later stimulation (-1.6 ± 0.5 dB for the checkerboard versus -2.75 ± 0.97 dB for the 3D- tunnel, (n = 8 subjects, *P* < 0.01 (ANOVA), d = 1.46, power = 0.87). In the central and frontal loci the ERD_max_ peaked at 200.0 ± 44.0 ms in case of the checkerboard stimulation and at 274.0 ± 29.0 ms for the 3D-tunnel and presented a significant difference in amplitude in favor of the latter stimulation (-1.1 ± 0.5 dB for the checkerboard versus -2.0 ± 0.68 dB for the tunnel, (n = 8 subjects, *P* < 0.008 (ANOVA), d = 1.4, power = 0.85). This difference was also highlighted in the ERSP map with the permutation test reaching statistical significance (*P* < 0.05) for the beta ERD between 250 ms and 500 ms (Fig 5C and 5F) and also 100 ms after the onset of the gray image for the central locus (Fig 6E). Taken all together these results indicated stronger neuronal excitation [58] when the 3D-tunnel was presented with respect to the checkerboard pattern (-3.5 ± 0.4 dB versus -1.2 ± 0.3 dB, *P* < 0.001, (ANOVA), d = 6.5, power = 1.66). The presence of a late theta ERS occurred mainly for the 3D-tunnel between 250 ms and 350 ms and was significantly different from the checkerboard stimulation (*P* < 0.05, permutation test) (Fig 5I).

Fig 6 illustrates the ERSP evoked by the gray image in the context of the checkerboard (*GrayCheck)* and the 3D-tunnel (*GrayTun*) in the same display as Fig 5. The statistical differences (permutation test) observed between the two situations consisted in the presence of a theta-alpha (4–8 Hz) ERS just after the arrival of the *GrayCheck* (Fig 6G and 6I) and not after the *GrayTun* (Fig 6H) indicating a relative re-synchronization of these slow rhythms after the checkerboard stimulation. In contrast, the existence of a stronger theta ERD in the occipital locus (-0.08 ± 1.08 for the tunnel versus 1.12 ± 1.02 dB for the checkerboard, (n = 12 subjects, *P* < 0.04, (ANOVA), d = 1.14, power = 0.86)(Fig 6G–6I), a stronger alpha and beta ERD in the central locus (Fig 6D–6F) and a stronger beta ERS at the latency of 450 ms for the *GrayTun* (n = 8 subjects, *P* < 0.03, (ANOVA), d = 1.2, power = 0.88)(Fig 6B, 6C, 6E and 6F) indicated increased activities after the tunnel presentation. These results were also confirmed by the FFT maps showing the scalp distribution of alpha (10 Hz) (Fig 6J) and beta (15 Hz)(Fig 6K) with significant differences in the centro-parieto-occipital electrodes and in the left centro-parietal electrodes (*P* < 0.05, permutation test), respectively.

### ERP generators

#### P100 generators

**Fig 7:**
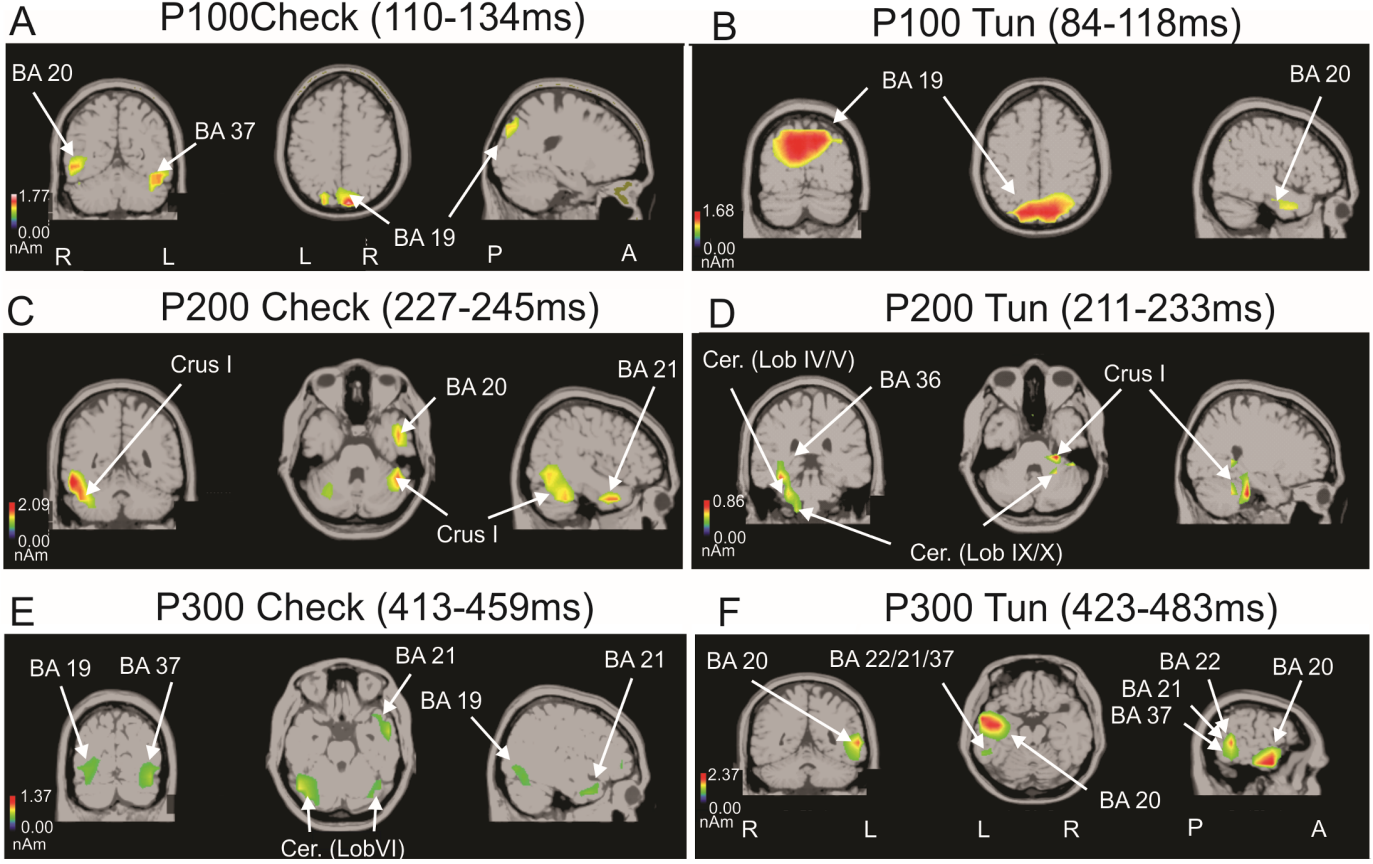
swLORETA sources obtained for P100, P200 and P300 event-related potentials (ERP) components. Nonparametric statistical maps calculated on all subjects for the checkerboard stimulation in relation to P100 (A), P200 (C) and P300 (E) and for the 3D-tunnel in relation to P100 (B), P200 (D), P300 (F). The white arrows point to the significant and respective ERP generators corresponding to Brodmann areas (BA) and specific regions of the cerebellum named in accordance to the Atlas of Human Cerebellum of (51)(see details in the main text).

swLORETA analysis performed on the ERP data (Fig 7) showed that the P100 generators were bilaterally localized in the cuneus of the occipital lobe (V2,V3-V5/MT) (BA18/19) (11(-18), -84, 31)(x, y, z coordinates) in the right inferior temporal gyrus (BA20) (53, -49, -9) and the left fusiform gyrus (BA37)(-46, -56, -17) for the checkerboard (Fig 7A). The same region of the occipital cortex (BA18/19) (13(-14), -84, 31) and the right inferior temporal gyrus (BA20) (42, -2, -25) were also identified for the 3D-tunnel (Fig 7B).

#### P200 generators

The P200 generators were maximally localized in the right inferior (BA20) (53, -45, -14) and middle temporal gyrus (BA21) (42, 2, -26) and the antero-lateral cerebellum (lobule VI/Crus I) mainly in the right (43, -41, -25) and accessory in the left side (-30, -51, -26) for the checkerboard (Fig 7C). For the 3D-tunnel, the P200 generators were localized in the right parahippocampal gyrus (BA36)(26, -22, -28), the right anterior lobe (culmen) (26, -39, -28)(lobule IV/V) and the right posterior lobe (tonsil) (18, -38, -40)(lobule IX/X) of the cerebellum (Fig 7D).

#### P300 generators

P300 generators were bilaterally localized in occipital (gyrus fusiform) BA19 (43 (-43), -70.5, -10.5) the posterior part of the cerebellum (40(-46), -58, -24)(lobule VI), the bilateral fusiform gyrus (BA37)(-57, -55, -8), the right middle temporal gyrus (BA21)(53, 2, -17) for the checkerboard stimulation (Fig 7E). For the 3D-tunnel, P300 generators were maximally localized in the left inferior temporal gyrus (BA20) (-48, -9, -18), the left middle temporal gyrus (BA21) (-55, -48, -1) and the left fusiform gyrus (BA37)(-58, -46, -16)(Fig 7F).

#### Gray image ERP generators

**Fig 8:**
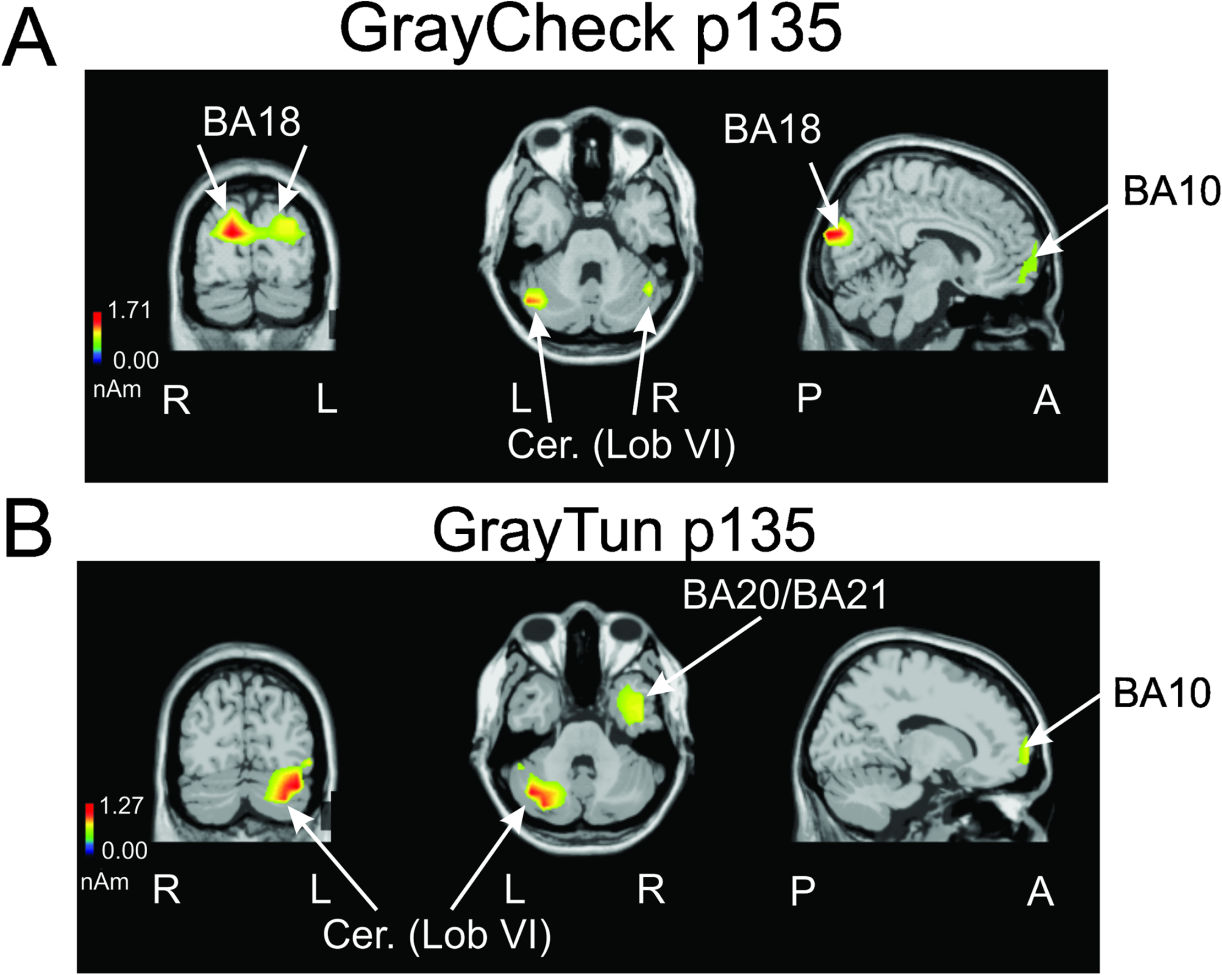
swLORETA sources, nonparametric statistical maps calculated on all subjects and obtained for the P135 component evoked by the gray image presentation in case of the checkerboard condition (GrayCheck)(A) and the 3D-tunnel condition (GrayTun)(B). The white arrows point to the significant and P135 generators corresponding to Brodmann areas (BA) and specific region of the cerebellum with the same nomenclature as used by (51) (see details in the main text).

swLORETA analysis performed at the P135 component latency evoked by the gray image presented between the checkerboard (*GrayCheck*)(Fig 8A) or between the 3D-tunnel (*GrayTun*) (Fig 8B) demonstrated that in spite of their uniform gray color, the right prefrontal cortex (BA10)(12, 62, 4) was commonly involved in both situations, P135 component evoked by the *GrayCheck* also involved the left prefrontal BA10 (-1, 59, 5), the bilateral V2 (BA18) (-27(18), -86, 20) and the left and right posterior part of the cerebellum (lobule VI) (46(-46), -61(-51), -25(-26); while the P135 evoked by the *GrayTun* also involved the left inferior temporal gyrus (BA20)(-57, -51, -11), the right middle temporal cortex (BA21)(49, -14, -14), the superior temporal cortex (BA22)(48, -14, -9) and the left posterior cerebellum (lobule VI) (-39, -64, -26).

#### The directional cue of the 3D-tunnel versus the scrambled version

In order to test the possible contribution of the directional change in the 3D-tunnel presentation, we compared the ERP, ERSP and generators when only the same direction (upward) of the 3D-tunnel were presented in 12 other subjects (*paradigm 2*, Fig 1B). We first confirmed the previous results obtained with the first group of subjects (*paradigm 1*, Fig 1A) concerning the basic differences between the checkerboard and the 3D- tunnel stimulation, namely smaller P100 and greater N200 for the 3D- tunnel with respect to the checkerboard (Fig 2).

**Fig 9:**
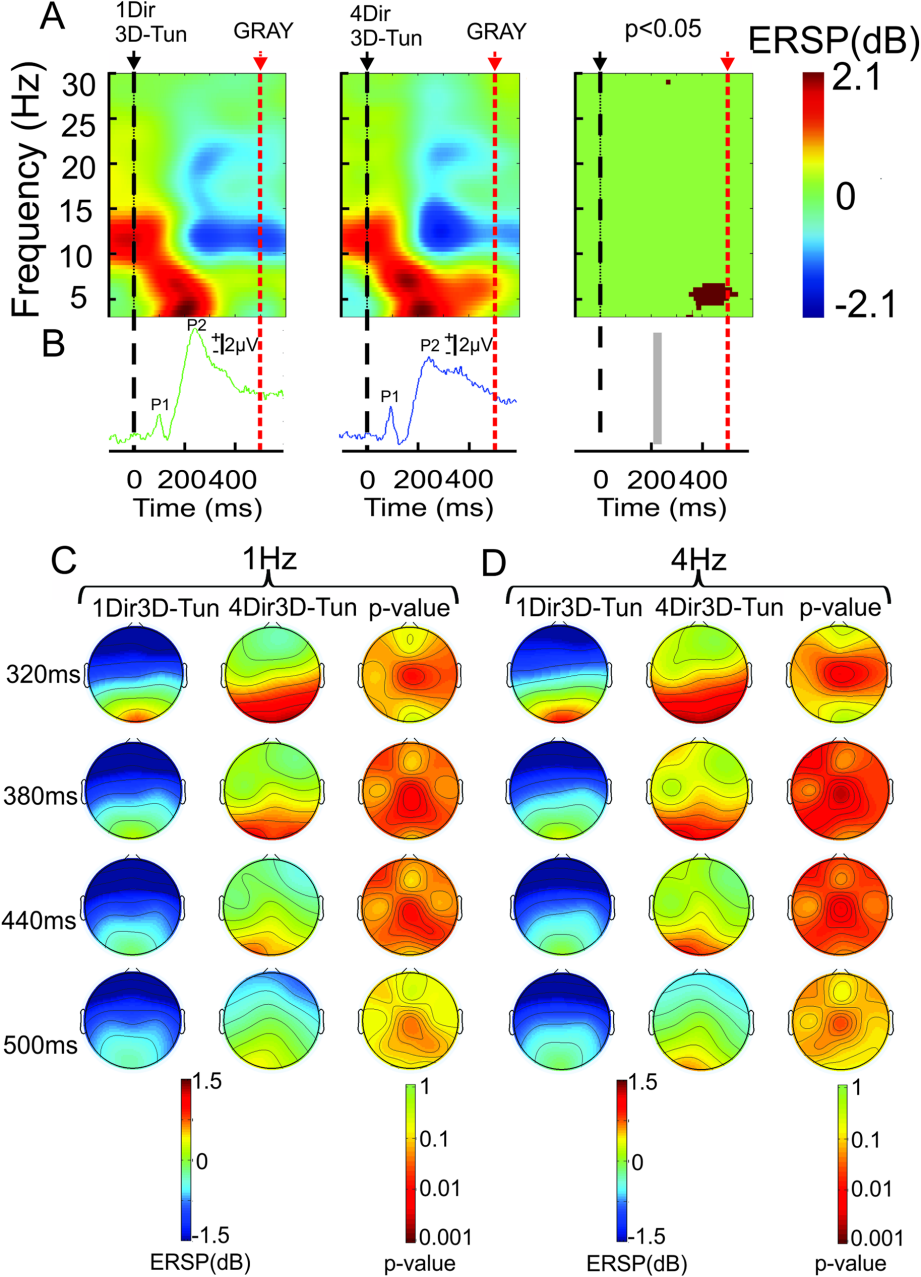
Effects of directional change in the 3D-tunnel presentation (grand average, n = 12 subjects). A: Event-related spectral perturbation in occipital locus (O2 electrode) evoked by the same 3D-tunnel direction (1Dir) (left side) and the 4 different directions (4Dir) (middle) showing a significant theta ERS between 370–500 ms for the 3D-tunnel with 4 different directions (middle map) with respect to the 3D-tunnel with single direction (left map). The statistical map (permutation with Holms test *P* < 0.05) is given on the right side of A. B: Event-related potential recorded (O2 electrode) evoked in the same condition as in A. The statistical difference (*P* < 0.05, permutation test) centered on the P200 component is marked by the vertical gray rectangle in the third column. C: Topography of the delta oscillation during the presentation of only one direction of the 3D-tunnel (left column) and during the 4 different directions of the 3D-tunnel presentation (middle column). The third column represents the statistical map (permutation with Holms). D: Same displays as in C but for the theta oscillation. Only the significant periods are illustrated.

The major differences observed between the single and multiple directions of the 3D-tunnel were expressed in the P200 component amplitude which was higher in case of the same direction (n = 12 subjects, *P* < 0.05 (permutation test))(Fig 9B) while in contrast, the late theta ERS was significantly stronger when multiple directions of the 3D-tunnel were used (n = 12 subjects, *P* < 0.05 (permutation test))(Fig 9A). This indicated the specific involvement of theta oscillation in directional viewing. In addition, the delta ERS oscillation and the absolute delta power (Fig 9C) were stronger at the P300 latency when the 4 different directions were used with respect to single direction of the 3D-tunnel. The topographical analysis showed that the theta ERS was significantly different (n = 12 subjects, *P* < 0.05 (permutation test)) for the electrodes situated in the centro-parieto occipital of the right side (Fig 9D).

**Fig 10:**
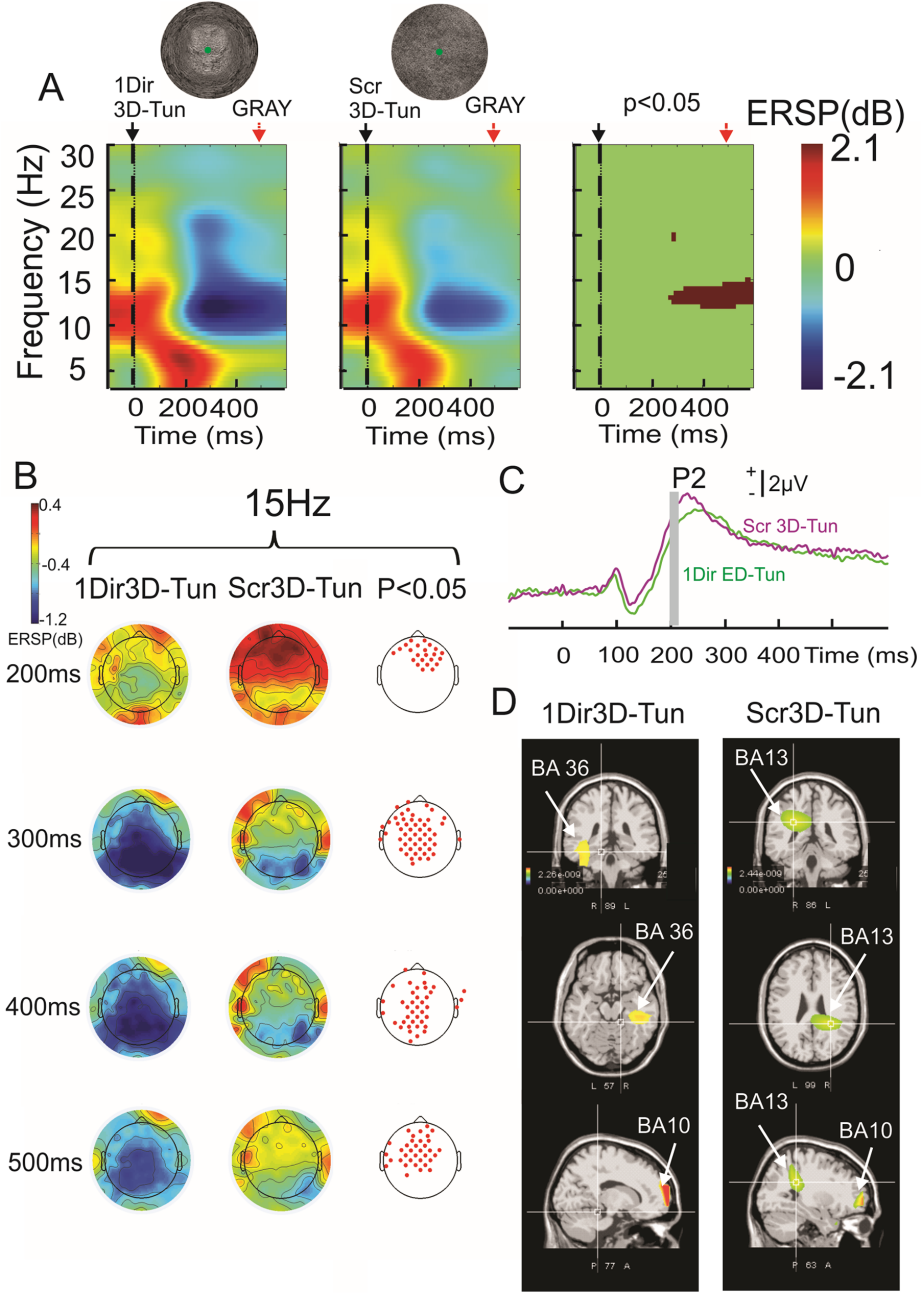
Comparison between the 3D-tunnel (1Dir3D-Tun) and its scrambled version (Scr3D-Tun)(grand average, n = 12 subjects). A: event-related spectral perturbation recorded at the parieto-occipital level (POz). Note the significant period in the alpha-beta ERD. B: Topography of the beta oscillation (15 Hz) during the 3D-tunnel (one direction)(first column) the scrambled version of the 3D-tunnel (middle column) and the related statistical map (permutation test, *P* < 0.05)(third column) from 200 ms to 500 ms. C: Superimposition of the ERP recorded at POz (3D-tunnel in green and scramble version in violet). D: swLORETA sources, nonparametric statistical maps calculated on all subjects (n = 12 subjects) and obtained for the P200 component evoked by the 3D-tunnel (left column) and the scrambled-3D-tunnel (right column). Note the contribution of right parahippocampal gyrus (BA36) for the 3D-tunnel and the right insular cortex (BA13) for the scramble version.

In order to better isolate the cognitive operations involved by the presentation of the 3D-tunnel, a scramble 3D-tunnel conserving the physical features but without a direction cue was compared with the “same direction” 3D-tunnel (*paradigm 3*, Fig 1C). This analysis (Fig 10) demonstrated a significant difference in the alpha-beta ERD between the 3D-tunnel and its scrambled version (Fig 10A). This difference occurred at the latency of 200 ms (Fig 10B) and persisted 100 ms after the end of image presentation. This power change was preceded by an increase of P200 amplitude in case of the scrambled-3D tunnel (n = 12 subjects, P < 0.05 (permutation test) (Fig 10C). When swLORETA was applied on the P200 component peaks (n = 8 subjects)(Fig 10D), the parahippocampal gyrus (BA36) already identified in the first group of 8 subjects during *paradigm 1* for the multi-directional 3D-tunnel (Fig 7D) was identified for the uni-directional (up direction) 3D-tunnel but not for the scrambled version of this image; while the prefrontal (BA10) was identified in both situations. Another prefrontal generator, located in right insular cortex (BA13) was found when the scrambled-3D-tunnel was presented (Fig 10D).

## Discussion

The present results demonstrate that the presentation of an image with action-oriented content of navigation (3D-tunnel) induces direction-specific ERP and ERSP changes in contrast to its scrambled version and to the presentation of a classical checkerboard image. The specificity of the 3D-tunnel content and the related top-down context induced stronger alpha-theta ERS anticipation in spite of the fact that the participants were in passive condition. In addition, this was followed by a stronger alpha-beta ERD indicating that visual input in VR may influence the brain states independently of the behavioral demand. In addition, our data shows that the related ERP generators identified by swLORETA point to the early (P200 latency) participation of the parahippocampal gyrus for the 3D-tunnel presentation.

### Differences in the ERP and ERSP configurations

The shorter P100 latency for the 3D-tunnel appears to be similar to previously reported shorter P100 latency for attended faces than for faces that needed to be ignored [59]. This was ascribed to a top-down effect.

In spite of the stronger P100 evoked by the checkerboard, the occurrence of an alpha ERS earlier prior to the presentation of the 3D-tunnel than the checkerboard, suggests a different top-down modulation for the 3D-tunnel. In the same line, the fact that the 3D-tunnel produced a stronger alpha-beta ERD after 200 ms that persisted after the occurrence of the gray image indicates a more intense recruitment of the neuronal activities implicated in the treatment of the 3D-visual image with respect to the checkerboard. This was also accompanied by a stronger theta phase locking at about 200 ms for the 3D-tunnel than for the checkerboard. As in the present condition, [20] reported a stronger beta ERD during action observation in experienced tennis players with respect to non-experienced players. The contribution of a stronger perception-action coupling of the mirror system in the experts was advanced for explaining the reinforcement of the ERD [20]. In the same line, the increased beta ERD for the 3D-tunnel observation may be viewed as resulting from the contribution of a perception-action coupling in VR navigation.

Changes in contrast may induce amplitude and/or latency shifts of P100 [60]. Therefore, stronger contrast in the checkerboard than in the 3D-tunnel may have induced greater phase-locking of the theta oscillation also explaining the higher P100 amplitude for the checkerboard presentation. Interestingly, experiments carried out in order to check for the effect of the direction of the 3D-tunnel (*paradigm 2*) demonstrated that the P200 amplitude is higher when the same tunnel direction is used (and repeated). This indicates that the changing of the 3D directional cue does not amplify the P200. In contrast, the fact that the directional change is accompanied by an increase of theta oscillation at P300 latency suggests that theta oscillation is related to directional recognition. This is in line with common acceptation that theta oscillation represents the ‘navigation rhythm’ [61–63], which is here already evoked by a virtual image representing directional turning and emerge preferentially on the right parieto-occipital cortex. The increase in delta oscillation favoring the P300 peak in the 3D-tunnel stimulation could be related to the high frequency activity (HFA, gamma 50–150 Hz) recorded by intracortical electrodes in the human inferior temporal cortex (BA20) [64] during visual working memory task, which could be recruited here due to the stimulus repetition [65]. The coupling between delta oscillation and HFA in the gamma band (~63 Hz) has been recognized during visual discrimination task in human MEG recordings [66] and was also recognized in the conscious access to visual target representation [67]. In this context, delta oscillation might represent the cyclical variations in the excitability of the neuronal pool represented by multiple unit activity (MUA) [68]. It may thus amplify the input signals and contribute to the persistence of the 3D-tunnel image after the occurrence of the gray image in the present case.

The possible influence of eye movement behaviors relating to the type of image can be ruled out as all the subjects respected the fixation requirement as indicated by the very small number of recorded saccades. Although fixation may facilitate the production of microsaccades and related gamma EEG activity [69], this is not relevant to the present results as they only concerned ERSP analysis under 30 Hz, i.e. far under the gamma oscillations related to saccades and microsaccades.

### ERP swLORETA sources

The fact that BA17 was not identified in the present study as a generator of P100 but rather BA18 and BA19 corroborates previous dipole modeling [70,71] showing that P100 represents a second stage in the cortical processing of visual input. This P100 component recorded in posterior loci presents a negative counterpart called here N100 in anterior loci when the earlobe, Cz or non-cephalic electrode were used as reference.

The most robust identification of bilateral V3 (BA19) as generator of P100 component is in accordance with the recent data of [72] demonstrating that human V3 is sensitive to rotation symmetry of texture stimuli (wall-paper pattern) at the P100 latency. While both types of stimulus involved P100 sources in bilateral V3 (BA19) and in the right inferior temporal gyrus (BA20), a region involved in visual working memory [64], the checkerboard recruits in addition the left fusiform gyrus (BA37) recognized by fMRI as taking part in the visual perception network (object recognition, visual language processing) [73], [74] and involved in picture encoding [75]. This additional generator situated in a high-level vision area of the ventral stream [76] may explain the larger amplitude of P100 in this condition. This may explain the preservation of P100 in weightlessness when the checkerboard stimulation was used in contrast to the alteration of P100 evoked by the 3D-tunnel [31]. Interestingly, BA37 was also involved in the generation of P300 (in case of the checkerboard) indicating long time involvement of this cortical area. Although P300 components were not evoked by a voluntary attention drift as in the classical oddball condition, swLORETA solution corroborated the LORETA solution obtained for face recognition described by [77] also pointing to BA19, BA37, BA20 and BA21.

The fact that the right parahippocampal gyrus (BA36) was only a generator for the P200 component evoked by the 3D-tunnel corroborates the implication of this region in spatial navigation [78] of the 3D-tunnel stimulation. However, the parahippocampal gyrus is also implicated in different aspects of visual processing, such as object location encoding [79], spatial configuration memory [80], familiar place [63], [81] and moving objects [82]. In addition, this region is anatomically connected to the visual associative cortex (V3 and V4 areas)[83]. When the 3D-tunnel presentation was compared with its scrambled version in a second group of 12 subjects, we confirmed the recruitment of the parahippocampal gyrus (BA36) previously identified in a first group of 8 subjects when the 3D-tunnel is presented. This reinforces the ability of 3D-tunnel image content to activate one of the main input regions of the hippocampal network implicated in navigation. The recruitment of the right insular cortex (BA13) and not BA36 when the scrambled version of the 3D-tunnel was presented may reflect implicit attempts by the subject to search for meaning in the scrambled image content, as highlighted by the demonstration of right BA13 activation in presence of logograms [15]. In addition, the more superficial localization of this generator with respect to the deeper BA36 source may explain the greater amplitude of P200 in favor of the scrambled-3D-tunnel.

The repetition of the same stimulus (checkerboard or 3D-tunnel) intermixed with a gray image points to the complex problems of priming, attenuation, adaptation and sensory suppression [84], [85] which are known to concern at least neuronal population of the inferior (BA20), medial temporal cortex (BA21) and fusiform gyrus (BA37), i.e. regions involved in the present ERP generators. While the repetition of familiar stimuli attenuated the neuronal response, repetition of unfamiliar stimuli enhanced the response in fusiform gyrus neurons [85].

### Contribution of the cerebellum

swLORETA analysis identified the contribution of different parts of the cerebellum, the right and left Crus I for the checkerboard and only right Crus I for the 3D-tunnel, and right cerebellar tonsil at the P200 peak latency. The latter cerebellar region has been recently implicated in processing of visual stimulation inducing nausea [86], a sensation that is occasionally reported by our subjects in response to the 3D-tunnel presentation. The nausea might also be the consequence of a conflicting situation where the body remains passive while the visual scene evokes movement. In addition, statistical mapping of cerebellar ischemic stroke, demonstrated that the tonsil is involved in the filtering of irrelevant visual information implicated in working memory task [87]. fMRI studies demonstrated that the cerebellum is involved in visual (passive) action observation [88], [89]. In particular, the left Crus I region is specifically involved in biological motion observation and functionally linked to the right superior temporal sulcus [27]. Surprisingly, we found here that the generators contributing to the P135 evoked by the *GrayTun* corresponded to a similar communicating network (left cerebellum and right superior temporal cortex) as the one reported by [27]. The fact that the implication of the cerebellum was identified in both types of image presentation (checkerboard or 3D-tunnel, for the P200 component) and the gray image (P135) may suggest that these cerebellar contributions are not related to the image content (except for the tonsil activation) but to the rhythmic aspect of the visual stimulation. In this context, the cerebellum may form a visuo-temporal template acting as a dynamic state estimator [90], as already demonstrated in a fMRI study [91]. As participants were asked to fixate a central mark during the visual stimulation, the eye position control exerted by the posterior cerebellum oculomotor vermis (lobules VI and VII) [92] and the lateral part of the cerebellum [93], both regions involved in saccadic control, could explain the present cerebellar contribution to the ERP generators.

## Conclusion

The present comparison of the brain evoked responses from two different visual patterns reveals specific wave features supported by common neural generators mainly for the P100 component (BA18,19,20) and more distinctive for P200 component highlighting the contribution of the parahippocampal gyrus (BA36) in the 3D-tunnel. This region implicated in navigation was also identified when the 3D-tunnel was compared with its scrambled version, highlighting a top-down effect linked to the navigational content. In spite of the richer detail content of the 3D-tunnel image with respect to the checkerboard, the parahippocampal gyrus contribution and the stronger delta-theta oscillation around the P300 component point to a prevalence of the effect of changing direction over the proper visual content of the 3D-tunnel. This may also underlie the prediction and the persistence of the 3D-tunnel with respect to the checkerboard revealed by the alpha-beta ERS and ERD, respectively. The cerebellar sources identified here by swLORETA for both images and gray pattern stimulations may corroborate the role of the cerebellum as a state estimator of the rhythmic visual pattern and eye fixation stability.
